# An automated assay platform for the evaluation of antiviral compounds against polioviruses^☆,☆☆^

**DOI:** 10.1016/j.jviromet.2024.115006

**Published:** 2024-08-08

**Authors:** Eric E. Rhoden, Bernardo A. Mainou

**Affiliations:** Polio and Picornavirus Branch, Division of Viral Diseases, Centers for Disease Control and Prevention, Atlanta, GA, USA

**Keywords:** Poliovirus, Coefficient of variation, Viral cytopathic effect (CPE), Cell cytotoxic infectious dose (CCID_50_)

## Abstract

High-throughput screening requires assays that have flexibility to test large numbers of specimens while being accurate to ensure reproducibility across all specimens and variables tested. Previously, we used a low-throughput, cell-based assay to identify compounds with antiviral activity against polioviruses. In this report, we report the development and implementation of a high-throughput automation platform for the identification of compounds with antiviral activity against polioviruses. The platform uses off-the-shelf automated equipment combined with a modified assay, with minimal changes to existing laboratory space. We evaluated automation systems from Hudson Robotics Inc., Agilent Technologies, and a microplate reader from PerkinElmer during the platform design. Optimization for high throughput was focused on bulk reagent additions, serial dilutions, microplate washing and measuring results from the tens-to-hundreds of microplates. We evaluated the automated cell-based assay for selectivity, sensitivity, accuracy, precision, and reproducibility. This platform can be applied to screen novel antivirals against polioviruses and non-polio enteroviruses.

## 1. Introduction

The Global Polio Eradication Initiative (GPEI) has relied almost exclusively on vaccines, including inactivated (IPV) and oral (OPV) poliovirus vaccines, to bring poliovirus to the brink of eradication (Sutter et al., 2008, Kew and Pallansch, 2018). In low pre-existing immunity settings, OPV can lose attenuating mutations and cause vaccine associated paralytic poliomyelitis (VAPP) in vaccine recipients and unimmunized contacts and evolve into endemic circulating vaccine-derived polioviruses (cVDPVs) (National Research Council, 2006). Once wild poliovirus transmission has been interrupted globally, OPV use will stop, leaving the IPV as the only tool to defend a polio-free world, according to the Global Action Plan for Poliovirus Containment (WHO, 2022). Immunocompromised individuals can be chronically infected with poliovirus and vaccines provide limited efficacy to control viral replication in this subpopulation (DeVries et al., 2011, Duintjer Tebbens et al., 2013). Small molecule inhibitors with antiviral properties against poliovirus could be used to complement IPV to treat infected individuals, including those that are immunocompromised, while also protecting those recently exposed to poliovirus, acting rapidly to contain outbreaks (De Palma et al., 2008a). Currently there are no approved anti-poliovirus drugs. However, experiences from drug development on related rhinoviruses and non-polio enteroviruses (Collett et al., 2008) and candidate drugs targeting polioviruses (Collett et al., 2017) point at the feasibility of developing drugs against members of the *Picornaviridae*. The National Research Council recommended that at least one, preferably two, poliovirus antiviral drugs be developed for control of polio outbreaks post-eradication (National Research Council, 2006) and some progress has been made (McKinlay et al., 2014, Stoyanova et al., 2024). In pursuit of this goal, our group implemented a cell-based assay to evaluate known compounds with potential antiviral activity against polioviruses.

Cell-based assays are routinely used as a quick, low-cost method to test compounds (Worzella, Larson, 2003, Van Loock et al., 2013, Ming et al., 2013). The general procedures include plating cells, dispensing, and equilibrating test reagents, adding compounds, incubating reaction mixtures, and quantitative assessment of cell viability. Our group has used a cell-based assay for compound screening against polioviruses similar to one used to define the antiviral properties of pleconaril against a panel of clinical enterovirus isolates (Pevear et al., 1999). Although this assay is reliable and cost-effective, it is labor-intensive due to repetitive steps needed for cell and reagent addition, serial dilutions, microplate washing, and quantification of cell viability using a plate reader. It is possible to apply robotic automation by modifying existing systems to increase testing throughput (www.hudsoncontrol.com). We explored the use of automation and modified our existing workflow to alleviate labor-intensive steps and increase throughput. Beyond increasing throughput, the automated platform needed to meet the following criteria: (1) use existing antiviral screening assay with minimal modifications; (2) use of stand-alone and exchangeable components; (3) use of equipment that fit into existing biosafety cabinets; (4) cost-effectiveness; (5) user-friendly hardware and software integration; and (6) readily expandable workflow for changes in testing volume. Here, we describe a modified poliovirus antiviral screening assay that integrates various instruments into an automated assay platform to increase testing throughput while also meeting our assay design criteria. We show that the automated assay generates data that is accurate, precise, reproducible, specific, and sensitive. This high-throughput assay will provide a tool to facilitate screening and identification of antiviral compounds against polioviruses with the flexibility to quickly adapt for testing antiviral compounds against other cytopathic viruses.

## 2. Materials and methods

### 2.1. MicroFlo^™^ select reagent dispenser

We used the MicroFlo^™^ Select Reagent Dispenser (Agilent Technologies) to dispense media and cells into microtiter plates. The system includes the MicroFlo^™^ Select reagent dispenser and microplate stacker (model BIOSTACKER2WR) with rotational wrist, 30-plate stackers, and a waste collection system. The MicroFlo^™^ Select programs allow for flexibility to select specific rows and columns on the multi-well plates for dispensing. The MicroFlo^™^ Select also has a small footprint that allows for placement into existing biosafety cabinets.

### 2.2. SOLO robotic workstation

A SOLO robotic workstation (Hudson Robotics Inc.) was used to perform virus and stock compound serial dilutions and transfer of diluted samples into assay plates. The system consists of a 12-channel robotic pipette assembly, landscape deck accessory kit with two nest holders, StackLink with two 30-plate capacity stackers, 24-inch Track-Link, a compressor supplying 80–120 psi compressed air supply, Solo-Soft (version 3.20) and SoftLinx (version 5.0.48) software, two StopLink microplate positioning devices, and an Edgeport 2-port USB-to-serial converter. The SOLO robotic workstation is controlled by a computer workstation. The SOLO robotic workstation has several advantages over other robotic systems that we evaluated, including its small footprint to allow for placement into existing biosafety cabinets, optional Stacklink plate stacker to provide 30-plate processing in a single run, and the SoftLinx software to integrate the Solo-soft programs into one simple procedure. The adjustable robotic pipette assembly can be configured in either landscape or portrait configurations, which allows for 12-channel or 8-channel serial dilutions in any direction.

### 2.3. EL406 microplate washer and dispenser system

For high volume liquid handling and washing of multi-well plates, we selected the EL406 microplate washer and dispenser (Agilent Technologies), paired with a microplate stacker (BIOSTACKER2WR) with rotational wrist and 30-plate stackers, and the Liquid Handling Control software (version 2) controlled by a computer workstation. The EL406 is connected to 10 L carboys for high volume liquid dispensing and waste collection. Waste collection allows downstream decontamination of any liquid components used when working with infectious agents. The computer workstation was used for facile programming of EL406, allowing nuanced protocol generation including delivery of liquids in various volumes and to specific areas of a microwell, liquid aspiration, and decontamination programs for various instrument components. The ability to adjust reagent delivery and aspiration to specific x, y, z coordinates within a microwell is essential for minimizing disruption of cell monolayers.

### 2.4. Victor X4 multi-mode plate reader

The Victor X4 multimode microplate reader (PerkinElmer) model 2030–0040 with a 595 nm absorbance filter was chosen to perform absorbance and luminescence readings. The Victor X4 was paired with two 40-plate stackers and a computer-controlled system running 1420 software (version 3.0) to allow high-throughput processing of assay plates.

### 2.5. Cell propagation and plate preparation

HeLa cells were obtained from CDC Scientific Resources Branch. A Bio-Rad automatic cell counter was used to enumerate cells and determine cell suspension dilution. One liter of cells (4×10^5^ cells/mL), when dispensed at 100 μL/well, was sufficient to seed 100 plates. The MicroFlo^™^ Select was used to dispense 100 μL cells in growth medium into 96-well, flat-bottom, polystyrene tissue-culture plates (Costar cat. No. 3598 or equivalent). Plates were wrapped in plastic wrap and incubated for 24 h at 37°C in a humidified 5 % CO_2_ incubator prior to use in the drug sensitivity assay.

### 2.6. Stock compound plate preparation

Stock solutions for compounds were made from freshly weighed compounds as 10 mM stocks diluted in high-performance liquid chromatography (HPLC)-grade dimethyl sulfoxide (DMSO, Sigma-Aldrich cat. No. 34869). Stock solutions were aliquoted in 500 μL aliquots in Wheaton 1.8 mL vials and stored at −80°C. On the day of the assay, vials of 10 mM compound stocks were thawed at ambient temperature (15 °C–25 °C) and further diluted to generate 2 mM working solutions in DMSO. A multichannel pipet was used to manually add 171 μL of DMSO to rows B through row H and 250 μL of 2 mM working compound solution was added to row A, columns 1–12, on a 96-well, flat-bottom polypropylene microplate (Corning cat. No. 7378). Using a multichannel pipet, 0.5 log_10_ serial dilutions of compounds were performed by transferring 79 μL of 2 mM compound in row A, columns 1–12, to row B columns 1–12 and performing subsequent 0.5 log_10_ dilutions through row G. There was no compound transferred to row H of the 96-well plate as this row serves as the virus only, no compound, control. Excess Stock Compound Plates can be stored at −80 °C for future use.

### 2.7. Preparation of compound dilution plates

**Fig. 1.**
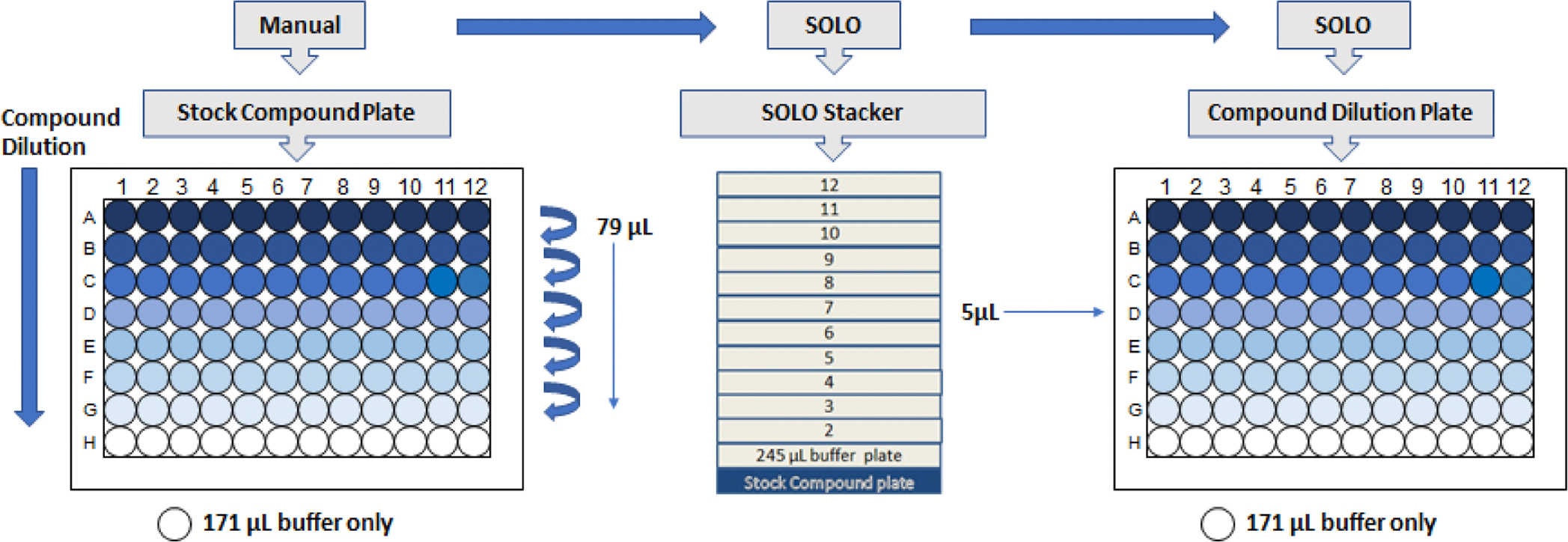
Preparation of Stock Compound and Compound Dilution Plate. A. 250 μL of 2 mM compound diluted in DMSO was added to row A. 171 μL of DMSO was added to rows B-H. Serial dilutions of 79 μL compound were performed from rows A-G. Row H contains DMSO only to serve as a vehicle control. B. The MicroFlo^™^ Select was used to add 245 μL assay buffer to all wells of a 96-well, flat-bottom plate (Compound Dilution Plate). The SOLO was used to transfer 5 μL of stock compound dilutions or DMSO alone from the Stock Compound Plate to the Compound Dilution Plate containing 245 μL assay buffer. Each Compound Dilution Plate of 250 μL (245 μL MEM+2 % FBS medium and 5 μL compound in DMSO or DMSO alone) is sufficient to overlay four cell-coated Assay Plates.

The MicroFlo^™^ Select added 245 μL of assay medium (e.g., MEM supplemented with 2 % Fetal Bovine Serum, FBS), to all wells of a 96-well, flat-bottom plate (Compound Dilution Plate). The SOLO was used to transfer 5 μL of DMSO or compound dilutions from the Compound Stock Plate (100 % DMSO) to the Compound Dilution Plate containing 245 μL of assay medium (2 % DMSO) (Fig. 1). Each Stock Compound Plate is sufficient to make 32 Compound Dilution plates. Each Compound Dilution Plate with a total volume of 250 μL per well is sufficient to overlay four Assay Plates (0.5 % DMSO).

### 2.8. Virus dilution plate preparation

**Fig. 2.**
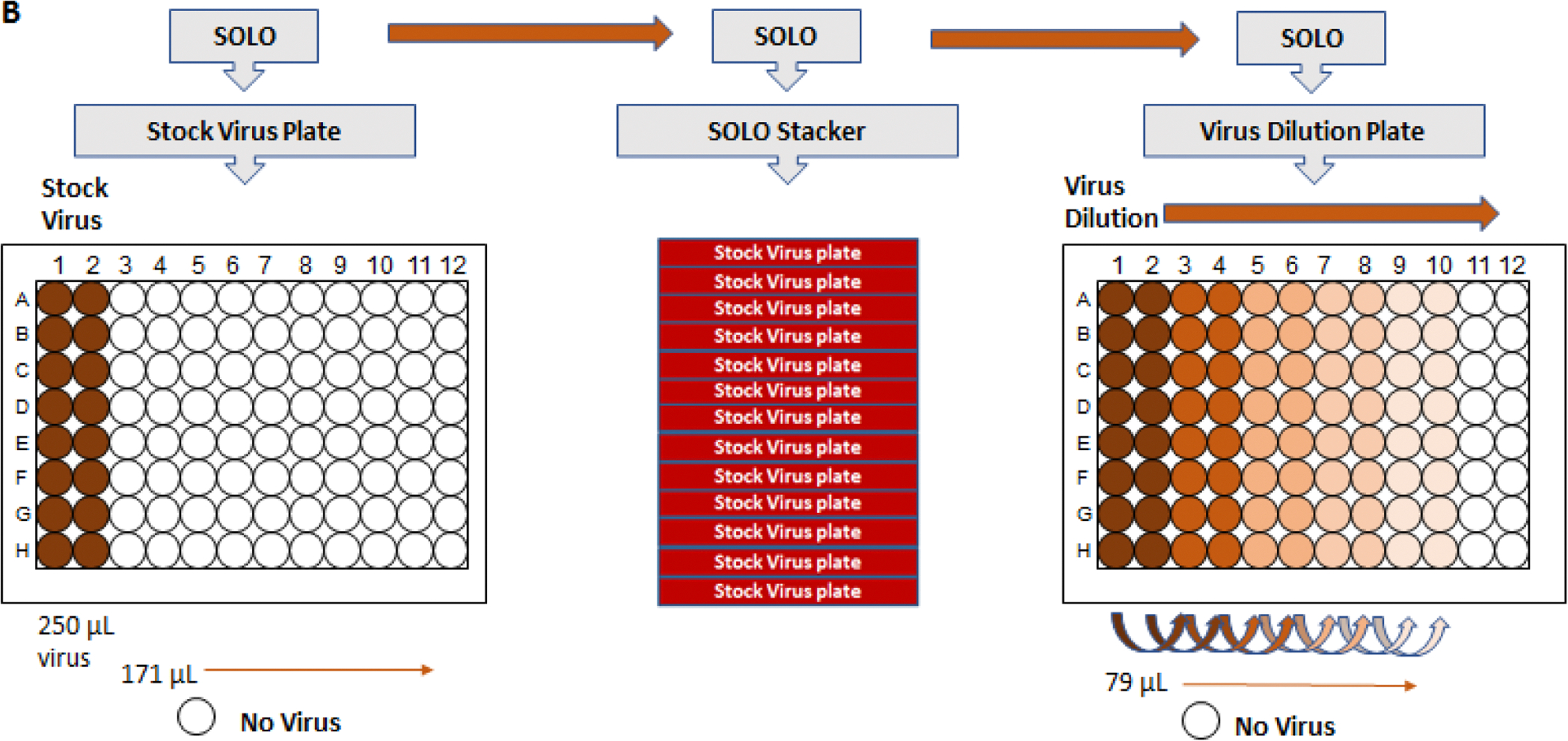
Preparation of virus dilution plate. The MicroFlo^™^ Select was used to deliver 171 μL of MEM+2 % FBS medium to all wells in columns 3–12 of 96-well flat-bottom plates. 250 μL of diluted virus stock was manually added to all wells in columns 1 and 2 of the plate. 12-point serial dilutions of stock virus were performed to determine starting CCID_50_ for use in the automated assay. We used the SOLO to perform a staggered 0.5 log_10_ dilution of the virus. Virus dilutions were performed by transferring 79 μL from columns 1–3, 3–5, 5–7 and 7–9. and repeated for columns 2–4, 4–6, 6–8 and 8–10. Columns 11 and 12 of the plate did not receive virus and served as mock-infected, assay compound controls.

One virus dilution plate is sufficient to inoculate two Assay Plates. A virus aliquot was thawed on ice and the MicroFlo^™^ Select was used to deliver 171 μL of MEM+2 % FBS medium to 96-well, flat-bottom polypropylene plates to all wells in columns 3–12. For each virus used, 12-point serial dilutions were performed to determine CCID_50_ concentrations for use in the automated assay. 250 μL of diluted virus at a concentration of 100 CCID_50_ were manually added to all wells of columns 1 and 2 of the plate. The SOLO was used to perform staggered 0.5 log_10_ dilutions of the virus by transferring 79 μL from columns 1–3, 3–5, 5–7, 7–9 and repeated with columns 2–4, 4–6, 6–8, 8–10. No virus is added to columns 11 and 12, with these columns acting as mock-infected, compound assay controls (Fig. 2).

### 2.9. Drug overlay and virus infection of assay plates

**Fig. 3.**
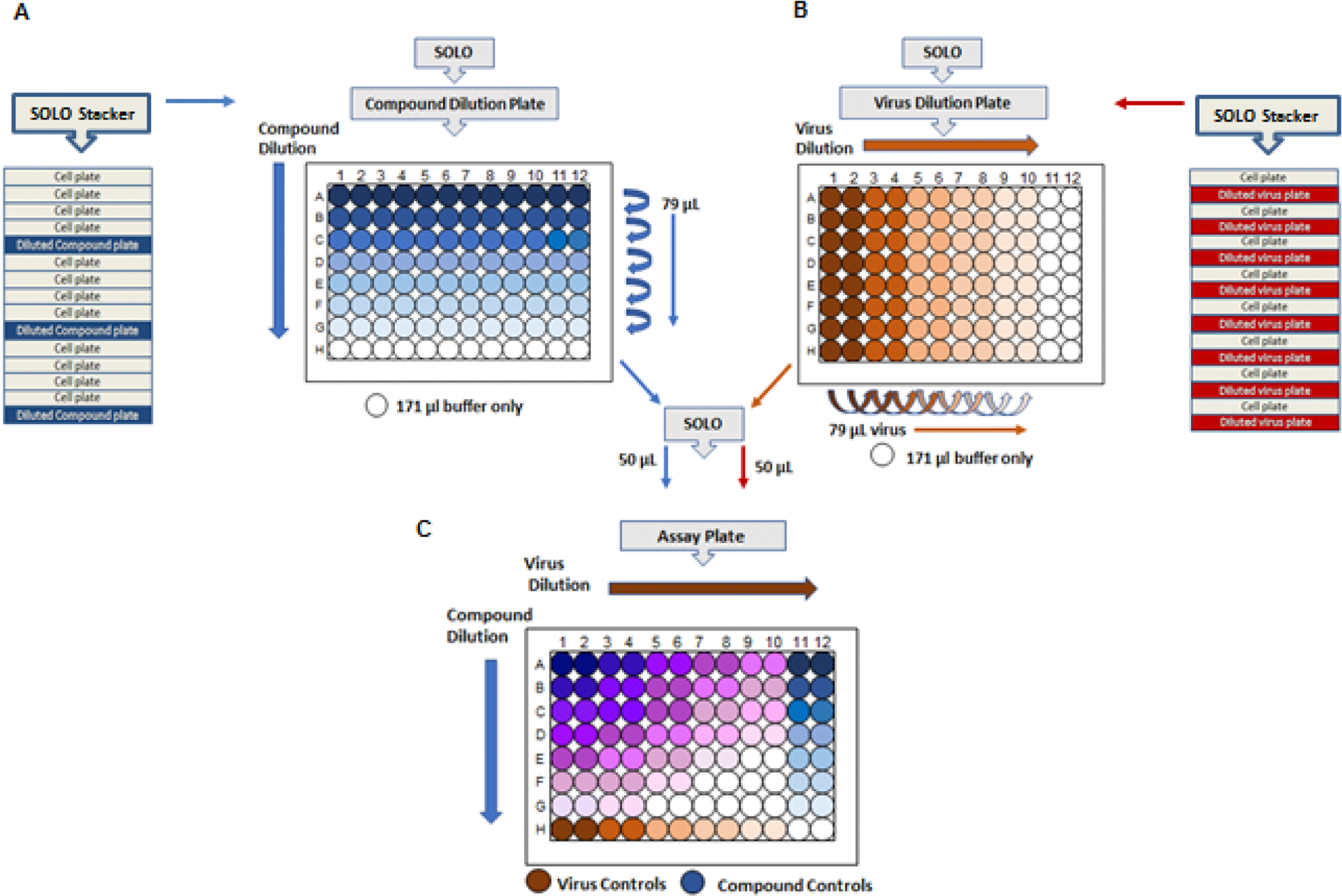
Preparation of antiviral screening assay plate. A and C. Assay (cell-coated) plates were microscopically examined to ensure presence of a confluent monolayer. The SOLO was used to transfer 50 μL of compound from the Compound Dilution Plate to each Assay Plate. Time between compound addition and virus addition is not to exceed 30 min to avoid undesired effects due to cells being exposed to higher compound/DMSO concentrations. The final DMSO concentration of 0.5 %, had no adverse effect on cells. B and C. The SOLO was used to transfer 50 μL of virus from the Virus Dilution Plate to the Assay Plate. Equipment movement was from most dilute to most concentrated to enabled use of the same tips for an entire Assay Plate. Completed Assay Plates were plastic wrapped and incubated in a humidified incubator at 37°C, 5 % CO_2_ for 3 days.

Before adding compounds, monolayer confluency in assay plates were checked under a microscope. The SOLO (Fig. 3) was used to transfer 50 μL of compounds from the Compound Dilution Plate and 50 μL of virus from the Virus Dilution Plate to the Assay Plate (Fig. 3). Time between compound addition and virus addition is controlled within 30 min to prevent adverse effects on cells resulting from higher concentrations of compound/DMSO (unpublished observation) and reduce assay variability. The final DMSO concentration of 0.5 %, had no adverse effect on cells. Instrument movement was programmed from most dilute to most concentrated, enabling the program to use the same tips for an entire cell plate (i.e., one virus per plate). Plates were then plastic wrapped and incubated in a humidified incubator at 37 °C, 5 % CO_2_ for 3 days.

### 2.10. Drug screening assay

The assay was adapted from an assay used to define the antiviral properties of pleconaril against a panel of clinical enterovirus isolates (Pevear et al., 1999). ViroDefense Inc. provided pocapavir, a capsid binder V-073 with known anti-poliovirus activity (Oberste et al., 2009) to serve as a positive control. CCL2 HeLa cells were seeded in 96-well plates (4×10^5^ cells/mL) in 100 μL growth medium (Minimal Essential Medium (MEM) with 2 % FBS) using the MicroFlo^™^ Select as described above and were incubated at 37 °C overnight. The following day, the SOLO was used to add 50 μL of diluted drug and virus in a cross-titration format (Fig. 3C). To ensure endpoints were reached for drug and virus titrations, the SOLO was used to perform five 7-point titrations in 0.5 log_10_ steps with duplicate wells for each drug-virus concentration. After three days incubation at 37 °C, plates were stained, using the EL406 microplate washer and dispenser, with crystal violet (0.05 % crystal violet, 0.5 % Tween-20, 50 % ethanol), followed by 3X washing with deionized water. The stained plates air dried overnight. Viral cytopathic effect (CPE) was determined by measuring absorbance at 595 nm using a Victor X4 plate reader.

An alternative procedure was used when weakly adherent or non-adherent cells were used. The incubation time and temperature selected were based on the virus type and cell line used (Zhang et al., 2019). ATPlite^™^ luminescence reagent (PerkinElmer) was used to measure intracellular adenosine triphosphate (ATP) as a proxy for cell viability (Miret et al., 2006). The MicroFlo^™^ Select reagent dispenser was used to add 50 μL of ATPlite^™^ cell lysis buffer then 50 μL of reconstituted substrate solution to each well. After a 10 min incubation at ambient temperature (15 °C–25 °C) in the dark, luminescence was assessed on a Victor X4 plate reader (PerkinElmer) configured with two 40-plate stackers and using 0.1 sec integration for each well. Raw luminescence counts were exported to a spreadsheet for analysis. This alternative approach is suitable for suspension cells and allows homogenous mix-and-measure assay format, which reduces liquid-handling steps for high-throughput assay. One limitation of this alternative approach is the assay requires a combination of cells and virus that results in CPE.

### 2.11. Data analysis

The percent inhibition values were analyzed by log(inhibitor) vs response-variable slope(4-parameter) curve-fitting and used to calculate the 50 % inhibitory concentration (IC_50_) for each compound (GraphPad Prism). A minimum of 3 independent experiments were performed on each virus-compound combination and the mean IC_50_ with standard deviation of the mean was reported. If virus was inhibited at all compound concentrations, the test was repeated starting at a 10-fold lower compound concentration.

## 3. Results

### 3.1. Assay performance

We set out to determine if our high-throughput method would meet or exceed the minimum standards recommended in the FDA Guidance for Industry Bioanalytical Method Validation www.fda.gov/regulatory-information/search-fda-guidance-documents/bioanalytical-method-validation-guidance-industry. The essential parameters required in the FDA Guidance are specificity, sensitivity, accuracy, precision, and reproducibility. We used these recommendations to evaluate the automated method used to screen compounds for potential poliovirus antiviral activity.

### 3.2. Specificity

**Fig. 4.**
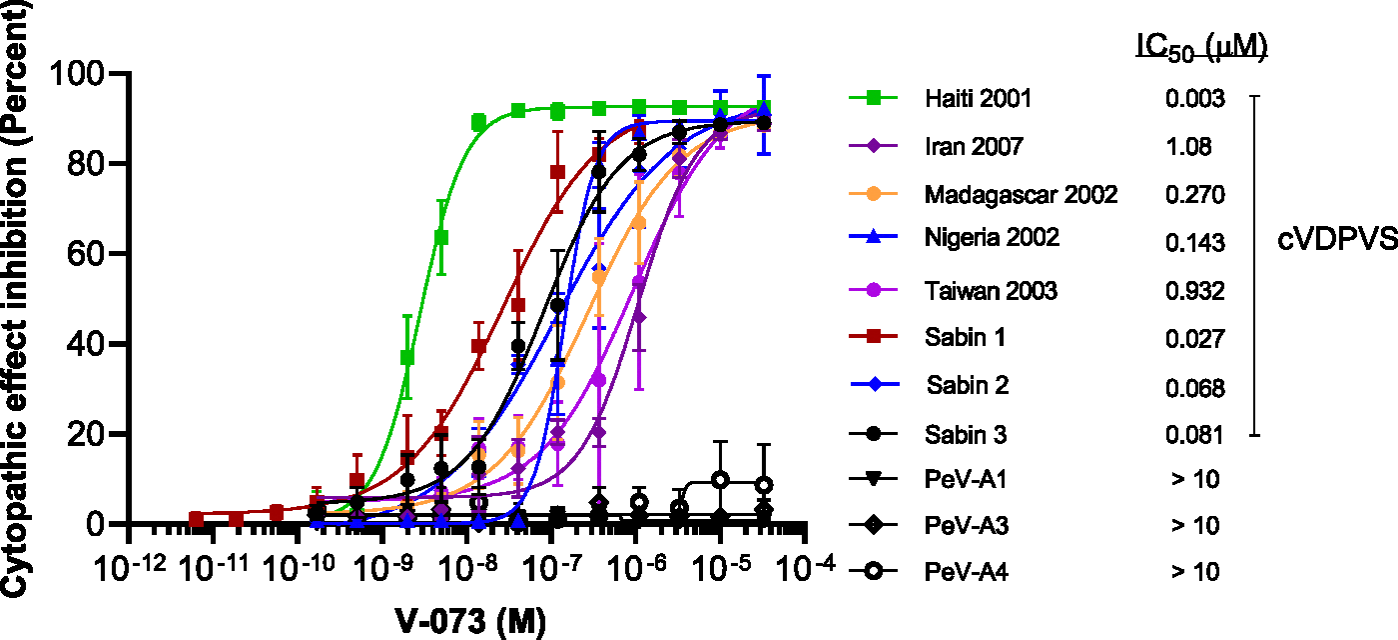
Specificity of antiviral screening assay. Representative poliovirus (PV) (Sabin 1, Sabin 2, Sabin 3, Haiti 2001, Iran 2007, Madagascar 2002, Nigeria 2002, and Taiwan 2003) and parechoviruses (PeV) (PeV-A1, PeV-A3, and PeV-A4) strains were tested against V-073 concentrations to determine the CPE in Hela cells. IC_50_s were calculated to quantitate the potency of the antiviral effect on susceptible virus strains. Data are shown as percent inhibition of CPE for three independent experiments.

From a panel of 45 diverse polioviruses, we selected a small subset and examined the assay specificity to identify and differentiate the activity of the V-073 (pocapavir), which has established antiviral properties against poliovirus (Oberste et al., 2009, Rhoden et al., 2013). These included Sabin Type 1 (Sabin 1), Sabin Type 2 (Sabin 2), Sabin Type 3 (Sabin 3), vaccine-derived polioviruses (VDPVs) Haiti 2001 (Type 1), Iran 2007 (Type 3), Madagascar 2002 (Type 2), Nigeria 2002 (Type 2), Taiwan 2003 (Type 1), and 3 parechoviruses (PeVs) PeV-A1, PeV-A3, and PeV-A4. The assay was able to distinguish the differing potencies of V-073 against the viruses tested, with a noted insensitivity to V-073 by all 3 parechoviruses tested (Fig. 4). Based on previous results (unpublished observation), it was expected that V-073 would not impact CPE induced by PeVs, thus serving as total negative (TN) controls (IC_50_ ≥ 10 μM).

Previous studies (Oberste et al., 2009, Rhoden et al., 2013), have showed that V-073 can block PV-induced CPE, thus acting as total positive (TP) controls (IC_50_ ≤ 10 μM). Total positive (TP) and total negative (TN) virus samples were compared to final positive (FP) and final negative (FN) virus samples to calculate percent specificity, using 50 replicates for each virus type (Table 1).

### 3.3. Sensitivity

**Fig. 5.**
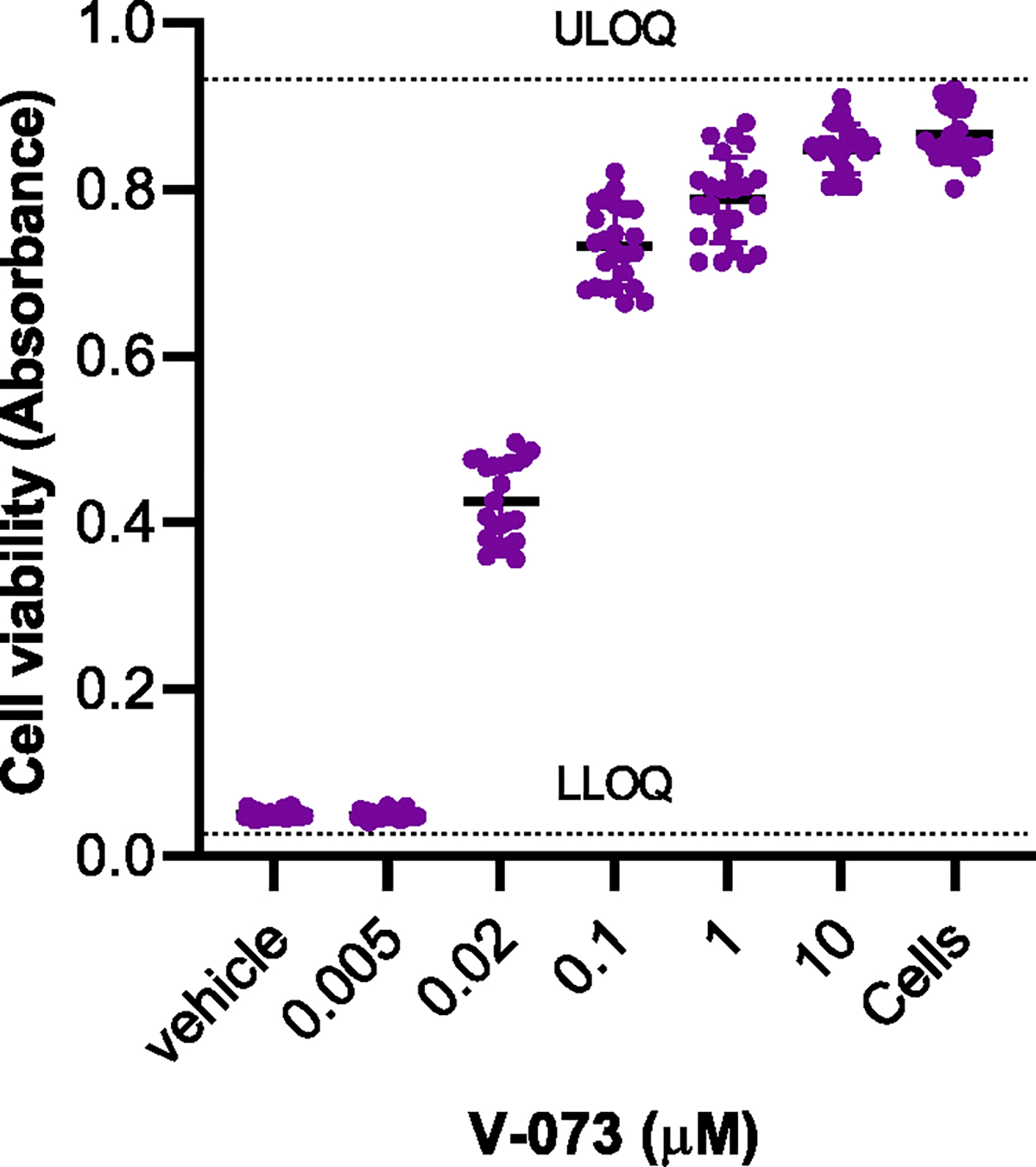
Effect of V-073 on Sabin 1 poliovirus induced CPE in HeLa cells. The ability of Sabin 1 poliovirus to induce cytotoxicity in HeLa cells was determined against increasing concentrations of V-073. Cell control wells (no virus) represent a 100 percent cell viability control. Vehicle control represents maximum CPE. Data are represented as cell viability (Absorbance at 595 nm) for each condition tested.

To determine the sensitivity of the assay, the ability of Sabin 1 PV to induce CPE was assessed against increasing concentrations of V-073 (Fig. 5). Results show the assay has an IC_50_ range of approximately 10 ^−8^ μM (lower limit of quantitation, LLOQ) to approximately 10 ^−5^ (upper limit of quantitation, ULOQ).

### 3.4. Accuracy

**Fig. 6.**
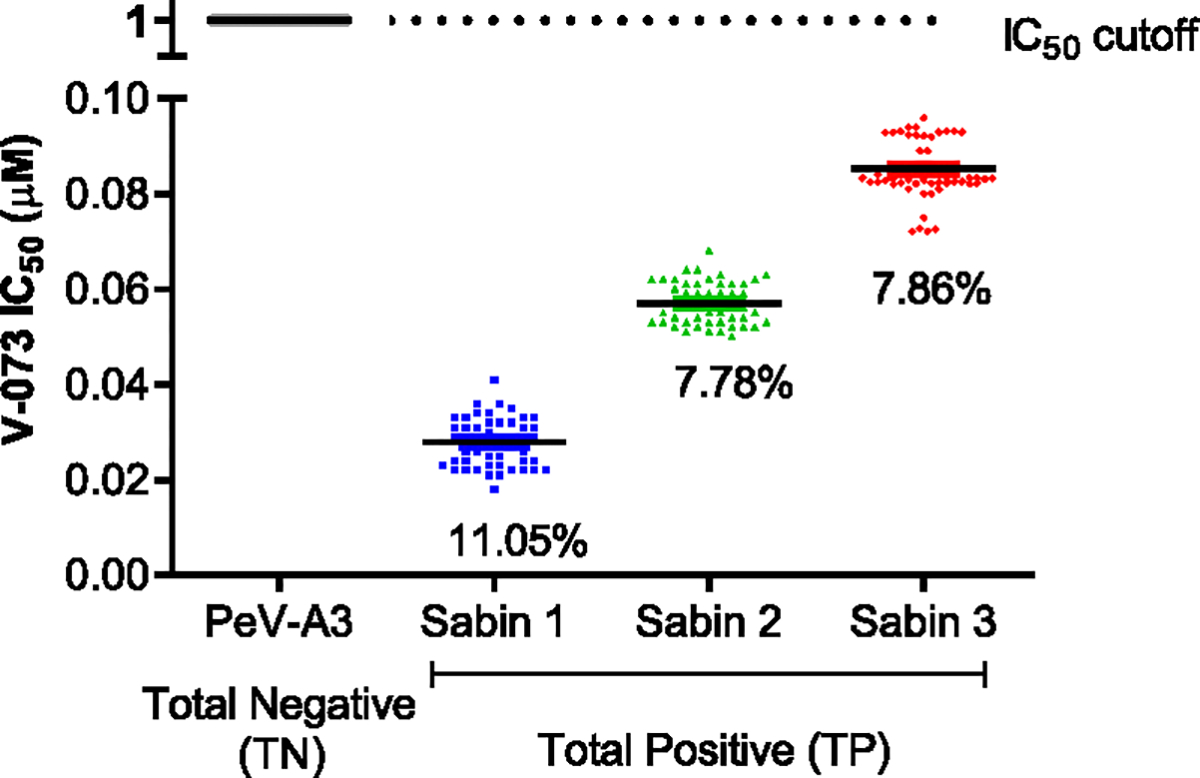
Accuracy of antiviral screening assay. Accuracy of IC_50_ measurement in total positive (TP) and total negative (TN) samples. IC_50_s from 50 Sabin 1–3 (TP) or PeV-A3 (TN) samples were tested in the presence of 0.01 – 0.2 μM V-073. The coefficient of variation (% CV) is shown for all three Sabin poliovirus serotypes. Data are represented as IC_50_ for each condition tested.

Assay accuracy was determined by replicate analysis of samples containing known concentrations of V-073 and calculating the percent coefficient of variation (%CV). FDA guidance recommends a minimum of three compound concentrations in a range of expected concentrations. Accuracy was measured using 50 replicates per compound concentration (0.01 – 0.2 μM V-073) to calculate inhibitory concentrations (IC_50_) against PeV-A3 and three Sabin poliovirus strains. The %CV was within 15 percent at all concentrations for all 3 Sabin poliovirus strains tested (Fig. 6). One PeV-A3 (TN) and three PV strains (TP) were tested. The %CV for the PV strains were 11.05 (Sabin 1), 7.78 (Sabin 2), and 7.86 (Sabin 3) respectively.

### 3.5. Precision

**Fig. 7.**
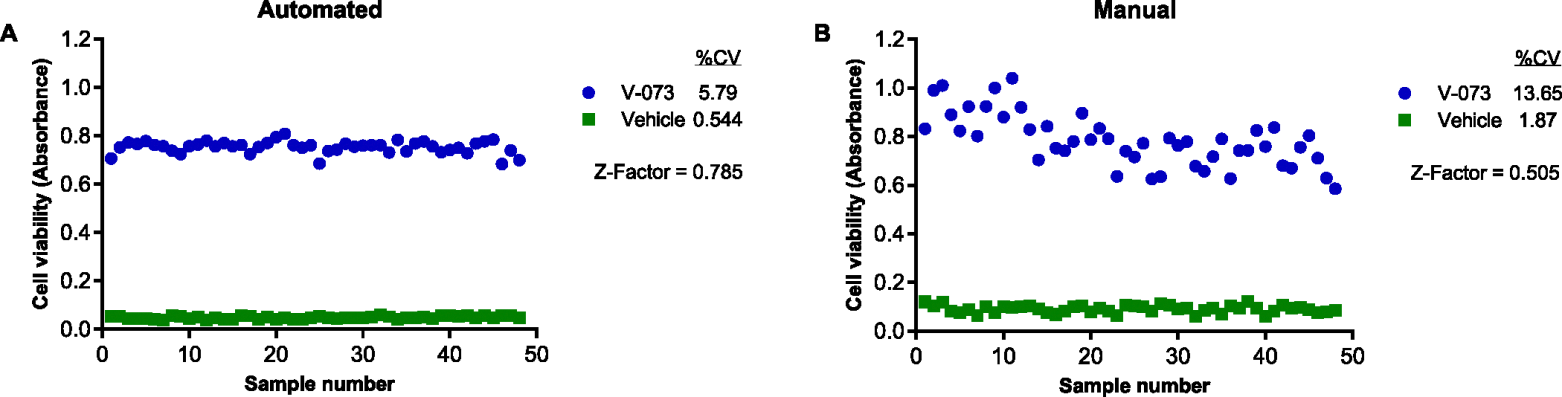
Precision and intra-assay reproducibility of automated and manual formats of antiviral screening assay. The automated (A) and manual (B) assay methods were assessed for precision by measuring the effect of vehicle control (DMSO) or 0.1 μM V-073 on Sabin 1. Data are represented as cell viability for 48 replicates for each condition.

Precision describes the closeness or scatter of individual measures of an analyte obtained for replicate samplings of a homogeneous sample. First, we measured precision as %CV. FDA guidance recommends the % CV should be within +/− 15 % at all concentrations except for the LLOQ, where it should not exceed 20 % of the CV. We used a maximal inhibitory concentration of 0.1 μM V-073 and Sabin 1 to compare the precision of the automated and manual methods (Fig. 7). The automated method yielded a %CV of 5.79 and the no drug control had a %CV of 0.544 (Fig. 7A). The manual method yielded a %CV of 13.65 and the no drug control had a %CV of 1.87 (Fig. 7B).

Next, we used Z-factor analysis (Zhang et al. 1999) as a measure of precision, reliability, and robustness for the assay. A Z-factor between ≥ 0.5 – 1.0 indicates that the values of the positive and negative controls are distant enough from each other for high assay reliability. The Z-factor for the automated was 0.785 compared to a Z-factor of 0.505 for the manual method (Fig. 7A, B), indicating a high level of precision, reliability, and robustness for the assay.

### 3.6. Reproducibility

**Fig. 8.**
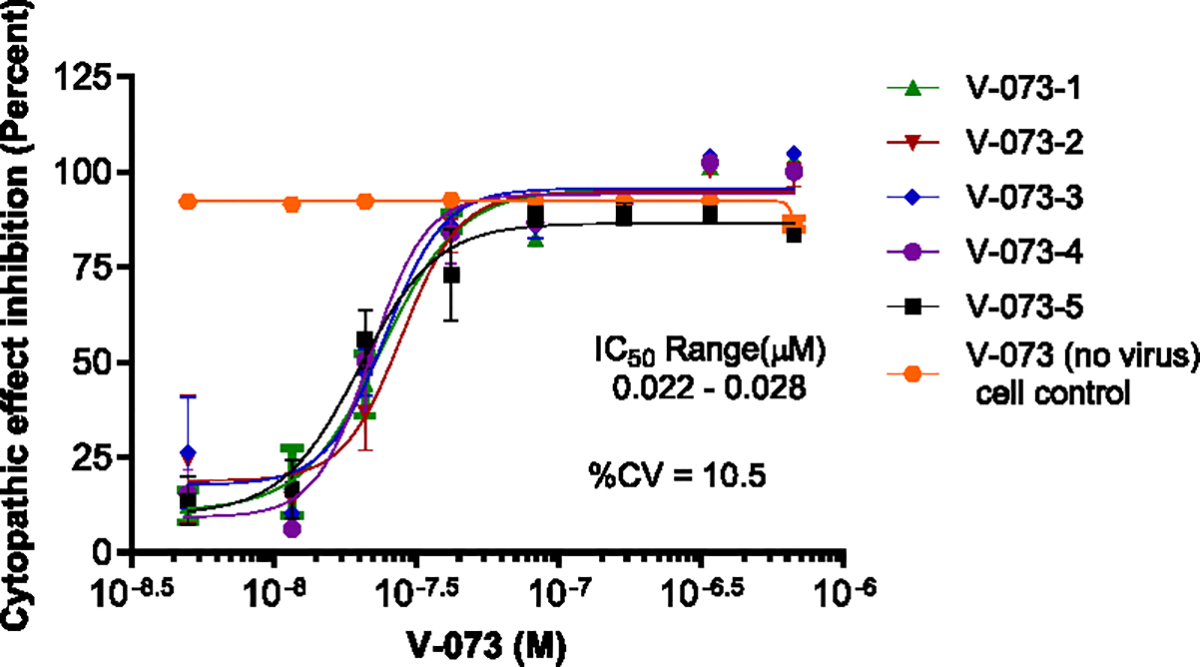
Intra-Assay Reproducibility. IC_50_ of V-073 against Sabin 1 was determined for replicate samples. Data represents five IC_50_ determinations performed on different days used to calculate %CV.

The reproducibility of the antiviral effects of V-073 on Sabin 1 was determined by performing repeat IC_50_ experiments (n=5) on different days (Fig. 8). The calculated %CV was 10.5 for the automated assay.

## 4. Discussion

We developed a high throughput poliovirus antiviral compound testing assay workflow by integrating automated equipment to facilitate bulk reagent additions, serial dilutions, microplate washing and data acquisition. We used FDA-established guidelines on selectivity, sensitivity, accuracy, precision, and reproducibility to evaluate the performance of our automated assay compared to the manual version. Our assay met the defined criteria for selectivity and sensitivity and the measurements for accuracy, precision and reproducibility were below the accepted criteria of 20 % CV.

The automated platform has been used to test compound antiviral activity against a panel of 45 polioviruses (Rhoden et al., 2013), to evaluate combination activity of compounds V-073 and V-7404 (Rhoden et al., 2013, McKinlay et al., 2014), a proof-of-concept study to assess the effectiveness of V-073 in healthy immunocompetent adults by evaluating virus excretion after administration of monovalent oral polio vaccine type 1 (mOPV1) (Collett et al., 2017) and for compassionate use, testing compound antiviral activity against immunodeficiency-associated vaccine-derived polioviruses (iVDPVs) from subjects (Israel 2017, Argentina 2017, Philippines 2019) (unpublished results). This automated assay platform has proven reliable for screening potential compounds against poliovirus (Rhoden et al., 2013, Tijsma et al., 2014) and has been used successfully in assessing the effectiveness of V-073 as a treatment for young children infected with enterovirus (Torres-Torres et al., 2015, Cataldi et al., 2015).

This automated assay platform increased testing throughput, while using existing laboratory space with minimal modifications. The automated assay exhibited better precision when compared to the manual assay, highlighting the benefit of implementing automation. The small footprint of the robotic instruments enables equipment installation in biosafety cabinets or containment laboratories with limited space when working on replication competent or high-risk viruses. The collection of liquid waste generated from the assay, via the direct connection of equipment to carboy containers, allows for efficient decontamination of infectious waste and proper disposal of chemical waste. The automated assay does have drawbacks, including significant capital investment to purchase instruments and annual maintenance costs, especially if multiple units are needed to perform all steps of the workflow. Cost-effectiveness is dependent upon the capability to sole source with multiple instrument manufacturers, the availability of personnel with automation expertize (for in-house method development, real-time troubleshooting, and instrument calibration), and the ability to procure bulk reagents and consumables.

Despite the relatively small footprint of the automated liquid handlers, once installed in biosafety cabinets or on laboratory benches, they cannot be easily moved, which limits the flexibility of using those spaces for other activities. This workflow is not limited for use with the selected instruments. The instruments can use reagents and consumables from a variety of manufacturers and it’s likely that assays optimized using different automated systems could achieve similar results.

Through increasing the automation of our antiviral screening assay, we have been able to alleviate labor-intensive steps such as serial diluting, bulk reagent additions, microplate washing and reading, while still achieving excellent precision and reproducibility. The modified high throughput testing platform described here is a useful tool for the identification of antivirals against poliovirus and other lytic viruses.

## Figures and Tables

**Table 1:** Specificity of antiviral screening assay using total positive (TP) and total negative (TN) virus samples.

Virus-Type	# Total positive (TP)	# Total negative (TN)	% Specificity TP/(TP+FN) × 100	% Specificity TN/(TN+FP) × 100

Haiti 2001–1	50	0	100	-
Iran 2007–3	50	0	100	-
Madagascar 2002–2	50	0	100	-
Nigeria–2002–2	50	0	100	-
Taiwan 2003–1	50	0	100	-
Sabin–1	50	0	100	-
Sabin–2	50	0	100	-
Sabin–3	50	0	100	-
PeV-A1	0	50	-	100
PeV-A3	0	50	-	100
PeV-A4	0	50	-	100

## References

[R1] Cataldi Jessica, Rhoden Eric, Oberste Steve, Hincks Jeffrey, Collett Marc, Wright Clyde, Asturias Edwin, 2015. Use of investigational antiviral drug pocapavir to treat enteroviral sepsis in twin neonates. Open Forum Infect. Dis. 2 (1), 478. 10.1093/ofid/ofv133.353.

[R2] Collett Marc S., Hincks Jeffrey R., Benschop Kimberley, Duizer Erwin, van der Avoort Harrie, Rhoden Eric, Liu Hongmei, Oberste M.Steven, McKinlay Mark A., 2017. Hartford Marianne, Antiviral activity of pocapavir in a randomized, blinded, placebo-controlled human oral poliovirus vaccine challenge model. J. Infect. Dis. 215 (3), 335–343. 10.1093/infdis/jiw542. 27932608 PMC5393058

[R3] Collett MS, Neyts J, Modlin JF, 2008. A case for developing antiviral drugs against polio. Antivir. Res. 79, 179–187. 18513807 10.1016/j.antiviral.2008.04.002

[R4] De Palma AM, Pürstinger G, Wimmer E, Patick AK, Andries K, Rombaut B, DeClercq E, Neyts J, 2008a. Potential use of antiviral agents in polio eradication. Emerg. Infect. Dis. 14, 545–551. 18394270 10.3201/eid1404.070439 PMC2570929

[R5] DeVries AS, Harper J, Murray A, Lexau C, Bahta L, Christensen J, Cebelinski E, Fuller S, Kline S, Wallace GS, Shaw JH, Burns CC, Lynfield R, 2011. Vaccine-derived poliomyelitis 12 years after infection in Minnesota. N. Engl. J. Med. 364 (24), 2316–2323. 10.1056/NEJMoa1008677. 21675890

[R6] Duintjer Tebbens RJ, Pallansch MA, Chumakov KM, Halsey NA, Hovi T, Minor PD, Modlin JF, Patriarca PA, Sutter RW, Wright PF, Wassilak SG, Cochi SL, Kim JH, Thompson KM, 2013. Expert review on poliovirus immunity and transmission. Risk Anal. 33 (4), 544–605. 10.1111/j.1539-6924.2012.01864. 22804479 PMC7896540

[R7] Food and Drug Adminstration. Guidance for industry: bioanalytical method validation.〈http://www.fda.gov/downloads/Drugs/GuidanceComplianceRegulatoryInformation/Guidances/ucm070107.pdf〉.

[R8] Kew O, Pallansch M, 2018. Breaking the last chains of poliovirus transmission: progress and challenges in global polio eradication. Annu. Rev. Virol. 5 (1), 427–451. 10.1146/annurev-virology-101416-041749. Epub 2018 Jul 12. 30001183

[R9] McKinlay MA, Collett MS, Hincks JR, Oberste MS, Pallansch MA, Okayasu H, Sutter RW, Modlin JF, Dowdle WR, 2014. Progress in the development of poliovirus antiviral agents and their essential role in reducing risks that threaten eradication. J. Infect. Dis. 210 (Suppl 1), S447–S453. 10.1093/infdis/jiu043. 25316866

[R10] Ming W, Ma W, Chen LH, Volk C, Michael MD, Xu Y, Zhang F, Wang X, 2013. Development and validation of a 96-well cellular assay for the discovery of ALDH1A1 inhibitors. Assay. Drug Dev. Technol. 11 (6), 388–395. 23957476 10.1089/adt.2013.513

[R11] Miret S, De Groene EM, Klaffke W, 2006. ‘Comparison of in vitro assays of cellular toxicity in the human hepatic cell line HepG2’. J. Biomol. Screen 11, 184–193. 16314402 10.1177/1087057105283787

[R12] National Research Council. 2006. Exploring the role of antiviral drugs in the eradication of polio. The National Academies Press, Washington, DC.

[R13] Oberste MS, Moore D, Anderson B, Pallansch MA, Pevear DC, Collett MS, 2009. In vitro antiviral activity of V-073 against polioviruses. Antimicrob. Agents Chemother. 53, 4501–4503. 19635956 10.1128/AAC.00671-09 PMC2764203

[R14] Pevear DC, Tull TM, Seipel ME, Groarke JM, 1999. Activity of pleconaril against enteroviruses. Antimicrob. Agents Chemother. 43, 2109–2115. 10471549 10.1128/aac.43.9.2109 PMC89431

[R15] Rhoden E, Liu HM, Wang-Chern SW, Oberste MS, 2013. Anti-poliovirus activity of protease inhibitor AG-7404, and assessment of in vitro activity in combination with antiviral capsid inhibitor compounds. Antivirus Res. 98 (2), 186–191. 10.1016/j.antiviral.2013.03.003 23499651

[R16] Stoyanova Adelina, Galabov Simeon, Galabov Angel S., 2024. Antiviral activity *in vitro* of double combinations of enteroviral inhibitors. Acta Virol. vol 68 10.3389/av.2024.12361.

[R17] Sutter RW, Kew OM, Cochi SL, 2008. Poliovirus vaccine live. In: Plotkin SA, Orenstein WA, Offit PA (Eds.), Vaccines Saunders. Elsevier, pp. 631–686.

[R18] Tijsma A, Thibaut HJ, Spieser SA, De Palma A, Koukni M, Rhoden E, Oberste S, Pürstinger G, Volny-Luraghi A, Martin J, Marchand A, Chaltin P, Neyts J, Leyssen P, 2014. H1PVAT is a novel and potent early-stage inhibitor of poliovirus replication that targets VP1. Antivir. Res. 110, 1–9. 10.1016/j.antiviral.2014.07.003. Epub 2014 Jul 17. 25043639

[R19] Torres-Torres S, Myers AL, Klatte JM, Rhoden EE, Oberste MS, Collett MS, McCulloh RJ, 2015. First use of investigational antiviral drug pocapavir (v-073) for treating neonatal enteroviral sepsis. Pediatr. Infect. Dis. J. 34 (1), 52–54. 10.1097/INF.0000000000000497. 25229269

[R20] Loock Van, Marnix Van den Eynde, Christel Hansen, John Geluykens, Peggy Ivens, Tania Sauviller, Sarah Bunkens, Lieve Van Acker, Koen Nijs, Erik Dams, Géry, 2013. An automated time-of-addition assay to routinely determine the mode of action of HIV-1 inhibitors. Assay. Drug Dev. Technol. 11 (8), 489–500, 10/2013. 24144343 10.1089/adt.2013.529

[R21] WHO Global Action Plan for Poliovirus Containment. 2022. (polioeradication.org/wp-content/uploads/2022/07/GPCAP-2022-2024.pdf).

[R22] Worzella T, Larson BA, 2003. Automating Promega cell-based assays in multiwell formats. Promega Corporation application note Number 85..

[R23] Zhang JH, Chung TD, Oldenburg KR, 1999. A simple statistical parameter for use in evaluation and validation of high throughput screening assays. J. Biomol. Screen 4, 67–73. 10838414 10.1177/108705719900400206

[R24] Zhang M, Zhang Y, Wang Y, Lv W, Zhang Y, 2019. Automated cell-based luminescence assay for profiling antiviral compound activity against enteroviruses. Sci. Rep. 9, 6023. 30988314 10.1038/s41598-019-42160-7 PMC6465263

